# Surface energy partitioning and evapotranspiration in a *Pinus tabuliformis* plantation in Northeast China

**DOI:** 10.3389/fpls.2023.1048828

**Published:** 2023-02-03

**Authors:** Xiang Gao, Jinsong Zhang, Jinfeng Cai, Songyi Pei, Linqi Liu, Ping Meng, Hui Huang

**Affiliations:** ^1^ Key Laboratory of Tree Breeding and Cultivation of National Forestry and Grassland Administration, Research Institute of Forestry, Chinese Academy of Forestry, Beijing, China; ^2^ Collaborative Innovation Center for Sustainable Forestry in Southern China, Nanjing Forestry University, Nanjing, China; ^3^ Henan Xiaolangdi Earth Critical Zone National Research Station on the Middle Yellow River, Jiyuan, China; ^4^ State-owned Jianping County Heishui Mechanized Forest Farm, Chaoyang, China

**Keywords:** surface energy partitioning, evapotranspiration, surface parameters, *Pinus tabuliformis* plantation, Northeast China

## Abstract

Examining the land-atmosphere interaction in vegetation rehabilitation areas is important for better understanding of land surface processes affected by human activities. In this study, energy flux observations were used to investigate surface energy partitioning and evapotranspiration (ET) in a *Pinus tabuliformis* plantation in Northeast China in 2020 and 2021. The sensible heat flux (H) was the dominant component of R_n_, and the ratio of H to the latent heat flux was higher than 1 at all growth stages. The two most important factors influencing the midday evaporative fraction and daily ET were the normalized difference vegetation index (NDVI) and soil water content at 10 cm depth (SWC_10_). Cumulative precipitation (P) minus ET was 62.83 and 239.90 mm in 2020 (annual P of 435.2 mm) and 2021 (annual P of 632.8 mm), respectively. The midday Priestley–Taylor coefficient (α), surface conductance (g_s_), and decoupling coefficient increased gradually from the onset of the mid-growing stage and decreased from the later growing stage. Midday α and g_s_ increased with NDVI and SWC_10_ increasing until the NDVI (0.5) and SWC_10_ (0.17 mm^3^ mm^−3^) thresholds were reached, respectively. Midday α and g_s_ were significantly influenced by vapor pressure deficit below 3 kPa, and the threshold value of midday g_s_ was approximately 12 mm s^−1^. In conclusion, this *Pinus tabuliformis* plantation regulated surface energy partitioning properly, and left a part of P for surface runoff and groundwater recharge in the semiarid region of Northeast China.

## 1 Introduction

Surface energy partitioning is an important object in the field of land surface-atmosphere interaction (Li et al., 2006; Gao et al., 2018), which controls hydrological processes in terrestrial ecosystems (Yan et al., 2017; Gao et al., 2021) and influences atmospheric circulation in the surface layer (Zhu et al., 2013; Jia et al., 2016). Many previous studies have demonstrated that the energy exchange between the atmosphere and the underlying surface is determined by vegetation characteristics and the climate system, which are closely linked to the thermodynamic process of the boundary layer (Hossen et al., 2011; Liu et al., 2019). Vegetation changes because of human activities could, in turn, change the surface energy budget and further influence the climate at local, regional, and even global scales (Lei and Yang, 2010; Kang et al., 2015). Therefore, considering climate change and vegetation rehabilitation, surface energy partitioning over vegetation rehabilitation areas must be comprehensively and precisely quantified to properly assess the effect of ecological restoration projects in Northeast China for addressing climate change.

Evapotranspiration (ET) is equal to the latent heat flux (LE) in an energy unit (Jia et al., 2016; Gao et al., 2021) and represents the second most substantial component of the hydrological cycle after precipitation (P) in the terrestrial ecosystem (Jung et al., 2010; Zhao et al., 2016). It links hydrological processes with energy exchange at the ecosystem scale (Zhu et al., 2013; Yan et al., 2017). Biophysical factors, such as solar radiation, soil moisture, vegetation type, and phenology, which control surface energy partitioning, also have an important effect on ET in various ecosystems (Zhu et al., 2013; Geng et al., 2020). The relative roles of biophysical factors are different in different ecosystem types; for example, ET is mainly controlled by air temperature (T_a_) and net radiation (R_n_) in tropical humid zones (Kuricheva et al., 2021). In contrast, the main influencing factors of ET in arid and semiarid areas are soil moisture and vapor pressure deficit (VPD) (Ji et al., 2021). Compared to rainfed croplands, grasslands, and deserts, lower albedo forests can absorb more solar radiation and thus partition more available energy for ET (Jia et al., 2016; Gao et al., 2021). Previous studies have shown that gross primary production is closely coupled with ET in terrestrial ecosystems, as leaf stomata simultaneously regulate photosynthesis and transpiration (Yuan et al., 2014; Gao et al., 2021). The water balance and carbon budget in vegetation rehabilitation are two major concerns for ecologists and policymakers (Kang et al., 2015; Gao et al., 2017) because of the mutual feedback between the hydrological and carbon cycles and climate change at different spatiotemporal scales (Gao et al., 2021). Since the implementation of the “Grain for Green Program” and “Three-North Shelter Forest Program”, many established plantations have altered land cover which has resulted in social, economic, and ecological benefits in Northeast China (Kang et al., 2015; Wang et al., 2021). Thus, there is an urgent need to investigate the seasonal variation in ET and its controlling factors for energy exchange process, hydrological cycle, and carbon sequestration in forests after rehabilitation.

Arid and semiarid ecosystems cover over 40% of the Earth’s land surface and are highly sensitive to land cover and climate change (Jia et al., 2016). Over 90% of the annual P in these ecosystems is lost to ET, and vegetation growth is often under water stress (Yuan et al., 2014; Zhao et al., 2016). With the implementation of ecological projects, many plantations of different tree species have been established for wind-breaking and sand-fixing in arid and semiarid regions of Northeast China (Wang et al., 2021). However, previous studies have indicated that some tree species used in the “Three-North Shelter Forest Program” are unsuitable for reforestation in arid and semiarid regions (Liu and Zhang, 2021; Wang et al., 2021). For example, poplar (*Populus* sp.) plantations, which require large quantities of water during the growing season, increase the risk of soil water deficit and threaten the long-term sustainability of arid and semiarid ecosystems (Kang et al., 2015). Furthermore, the climate is becoming warmer and drier in arid and semiarid regions, and the conflict between plantations and water may worsen in Northeast China (Lian et al., 2021). Therefore, to optimize the management and construction of plantations in arid and semiarid regions of China under climate change, investigating the surface energy partitioning and ET of plantations of different tree species is crucial.

*Pinus tabuliformis* is widely used in the “Three-North Shelter Forest Program” because of its resistance to drought and ability to grow in barren soil (Zhao et al., 2021). *Pinus tabuliformis* plantations, covering an area of 167.76 × 10^4^ ha, play key role in water and soil conservation and carbon sequestration in northern China (Zhao et al., 2021). The arid and semiarid regions of Loess Plateau and Northeast China are two typical planting areas of *Pinus tabuliformis* in China. Some studies found that *Pinus tabuliformis* plantations could cause deep soil drying, which resulted in sub-healthy plantations on the Loess Plateau (Li et al., 2010). The natural environment on the Loess Plateau is entirely different from that in Northeast China, and little attention has been paid to the effect of *Pinus tabuliformis* plantations on water and energy cycle in Northeast China. Therefore, investigating water vapor and surface energy fluxes and their dominant influences is vital for ascertaining the growth status of *Pinus tabuliformis* plantations and their possible changes under warmer and drier climates in the arid and semiarid regions of Northeast China.

Given the above considerations, year-round energy fluxes, ET, and related biophysical data were collected in 2020 and 2021 in a *Pinus tabuliformis* plantation in the semiarid region of Northeast China. The objectives of this study were to: (1) characterize the seasonal variations in surface energy partitioning; (2) investigate seasonal variation in ET and examine the water balance; and (3) determine the surface parameters characterizing surface energy partitioning and ET. In particular, we checked the effects of biophysical factors on evaporative fraction (EF), ET, and surface parameters. We hypothesized that canopy growth and soil water status exert the most important control on surface energy partitioning, water vapor loss, and surface development in the *Pinus tabuliformis* plantation.

## 2 Materials and methods

### 2.1 Study site

**Figure 1:**
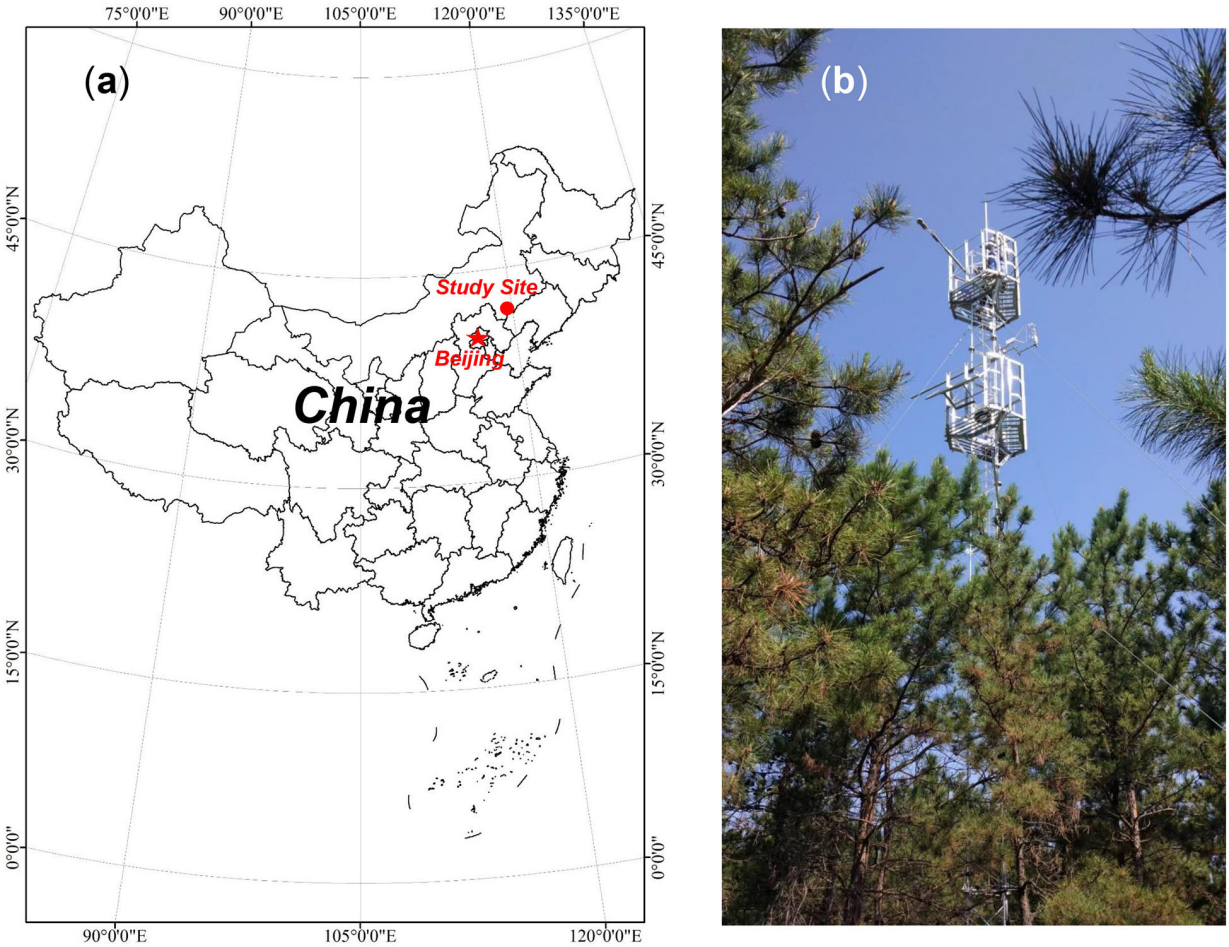
Location of the study site **(A)** and the observation tower **(B)**.

The study site is located in the State-owned Jianping County Heishui Mechanized Forest Farm (42°6′34′′ N, 119°29′57′′ E, 650 m a.s.l.), Chaoyang City, Liaoning Province, Northeast China (**Figure 1A**). The area has a semiarid temperate continental monsoon climate with a mean annual temperature of 5.76°C. Mean annual evaporation and sunshine duration are 1,962.10 mm and 2,922 h, respectively. The yearly mean P is 440 mm, with 67% rainfall occurring from June to August. The soil type at the site is grey-brown, with a soil bulk density of 1.31 g cm^–3^ and a field capacity of 0.32 cm^3^ cm^–3^. The *Pinus tabuliformis* plantation is 36 years old with a density of 1,500 stems ha^–1^, an average tree trunk diameter at chest height of 11.8 cm, and a canopy height of approximately 8.0 m. The understory covers approximately 80% of the site and is dominated by *Lespedeza bicolor*, *Carex dispalata*, and *Cleistogenes polyphylla*.

### 2.2 Measurements

**Figure 2:**
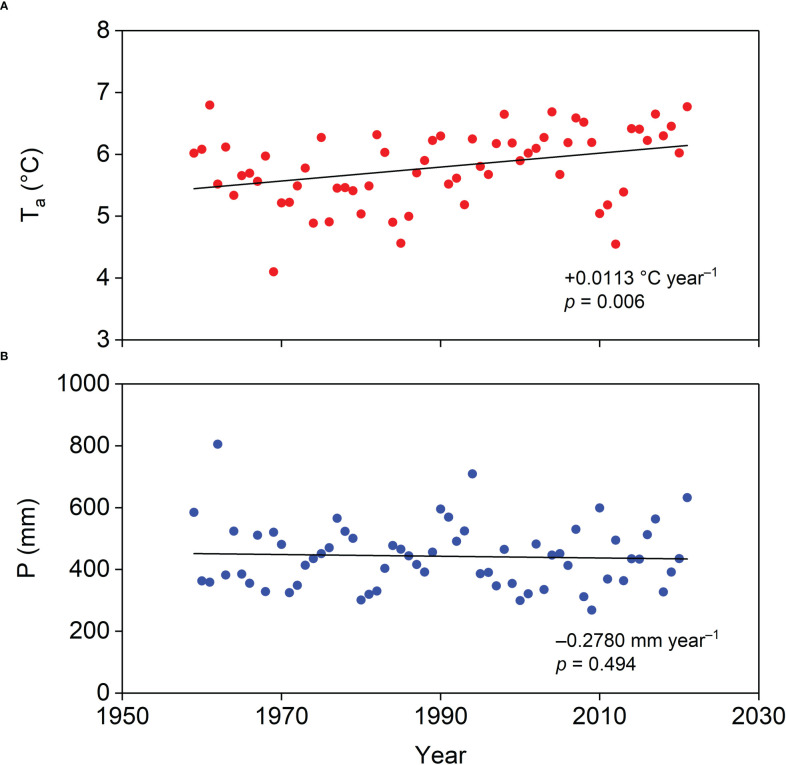
General trends in **(A)** annual mean air temperature (T_a_) and **(B)** annual precipitation (P) in our study area during 1959–2021.

An 18 m tower was erected on the plantation to mount instruments for capturing biophysical and energy flux measurements (**Figure 1B**). The instrument details are listed in **Table 1**. A data logger (CR1000, Campbell Scientific Inc., Logan, UT, USA) was used to record the data measured by the instruments on the tower. The distance from the tower to the nearest boundary of the plantation was approximately 300 m, ensuring that the measured signal originated from the plantation. The annual P and annual mean T_a_ between 1959 and 2021 were collected from the Jianpingzhen National Meteorological Station (41°52′ N, 119°38′ E, 662 m a.s.l.), 30 km from the tower. Trends in annual P and mean T_a_ from 1959 to 2021 are shown in **Figure 2**. The normalized difference vegetation index (NDVI) time series of the plantation was obtained from the 250 m multi-temporal MODIS NDVI 16-day composite data (https://ladsweb.modaps.eosdis.nasa.gov/). ArcGIS 10.6 software (ESRI Inc., Redlands, CA, USA) was used to extract the pixel value within the plantation area.

**Table 1:** List of measured items and environment monitoring instruments installed on the tower in the *Pinus tabuliformis* plantation.

Observations	Height/depth (m)	Model, manufacturer	Accuracy
Latent (LE) and sensible heat flux (H), fraction velocity (u_*_), wind speed (U)	12	LI-7500, Li-COR Inc., Lincoln, NE, USA^a^	± 1%
	12	CAST3B, Campbell Scientific Inc., Logan, UT, USA^a^	< ± 2%
Air temperature (T_a_) and relative humidity (H_a_)	12	HMP45C, Vaisala Co., Ltd., Helsinki, Finland	± 0.2°C/3%
Downward (S_d_) and upward shortwave radiation (S_u_), downward (L_d_) and upward longwave radiation (L_u_)	16	CNR4, Kipp&Zonen B.V., Delft, Netherlands	<1%
Soil water content^b^	0.1; 0.3^c^	CS650, Campbell Scientific Inc.	± 1%
Soil heat flux (G)^b^	0.1	HFP01SC, Hukseflux B.V., Delft, Netherlands	± 2%
Precipitation (P)^d^	16	TE525MM, Texas Electronics Inc., Dallas, TX, USA	0.1 mm

a eddy covariance system consisting of a 3D sonic anemometer (CSAT3B) and an infrared H_2_O/CO_2_ gas analyzer (LI-7500).

b installed around the tower, and 3 repetitions at each depth.

c depth referring to a previous study (Cheng and Chen, 2013).

d calibrated from the Jianpingzhen National Meteorological Station, 30 km from the tower.

The growing season (GS) of the plantation was divided into three phenological stages based on the growth rhythm of *Pinus tabuliformis*, namely the early growing stage (EG), mid-growing stage (MG), and later growing stage (LG). The germination and pine needle elongation period is represented by EG, the flourishing period by MG, and the pine needle senescence period by LG. According to manual records, EG extended from April to May, MG extended from June to September, and LG occurred during October. The remaining months comprised the dormant season (DS).

### 2.3 Data processing

Eddypro 7.0.7 (Li-COR Inc., Lincoln, NE, USA) software was used for calibration and quality control of the 10 Hz raw data and generated 30-min data. In addition, 21.27% of the LE and 30.31% of the sensible heat flux (H) values were rejected because of quality control and equipment failure. For missing LE and H data, short gaps (≤ 2 h) were filled using a linear relationship, and lengthy gaps (> 2 h) were filled using the mean diurnal variation (MDV) method (Falge et al., 2001).

The net shortwave and longwave radiations (S_n_ and L_n_) and net radiation (R_n_) were calculated as follows:


$$S_{n}=S_{d}-S_{u}$$

$$L_{n}=L_{d}-L_{u}$$

$$R_{n}=L_{n}-S_{n}$$

where S_d_ and S_u_ are the downward and upward shortwave radiation, respectively, and L_d_ and L_u_ are the downward and upward longwave radiation, respectively. The ET was calculated as follows:


$$ET=\frac{LE}{\lambda }$$

where *LE* is latent heat flux (W s^−1^), and *λ* is the latent heat of the vaporization of water (2.45 kJ g^−1^). The evaporative fraction (EF) was calculated as follows:


$$EF=\frac{LE}{R_{n}}$$

The crop coefficient (K_c_) was calculated according to Allen et al. (1998) as follows:


$$K_{c}=ET/ET_{0}$$

$$ET_{0}=\frac{0.408\Delta \left(R_{n}-G\right)+900\cdot U\cdot \gamma \cdot VPD/\left(T_{a}+273.3\right)}{\Delta +\gamma \left(1+0.34U\right)}$$

where ET_0_ is the reference evapotranspiration, U is the wind speed (m s^−1^), VPD is the vapor pressure deficit (kPa), Δ is the slope of the water vapor pressure curve (kPa °C^−1^), and γ is the psychrometric constant (kPa °C^−1^).

The aerodynamic conductance (g_a_) was estimated according to Monteith and Unsworth (1990), and surface conductance (g_s_) was calculated by inverting the Penman–Monteith equation (Allen et al., 1998):


$$g_{a}={\left(\frac{U}{U_{*}^{2}}+6.2U_{*}^{-2/3}\right)}^{-1}$$

$$g_{s}=\frac{\gamma \cdot LE\cdot g_{a}}{\Delta \left(R_{n}-G\right)+\rho_{a}\cdot c_{p}\cdot VPD\cdot g_{a}-LE\left(\Delta +\gamma \right)}$$

where U_*_ is the friction velocity (m s^−1^), ρ_a_ is the air density (1.2 kg m^−3^), and c_p_ is the specific heat of the dry air (1,004.7 J kg^−1°^C^−1^). The decoupling coefficient (Ω) was introduced to quantify the sensitivity of LE to g_s_ (Monteith and Unsworth, 1990). When g_a_ is close to +∞, Ω is close to 0, which means that the underlying surface is well coupled with the environmental conditions. Under this condition, the Penman–Monteith equation is transformed to:


$$LE\to LE_{im}=\left(\frac{\rho_{a}c_{p}}{\gamma }\right)VPDg_{s}$$

where LE_im_ is the imposed latent heat flux (W s^−1^). When g_a_ is close to 0, Ω is close to 1, indicating that the underlying surface is poorly coupled with the environment. Under this condition, the Penman–Monteith equation is transformed to:


$$LE\to LE_{eq}=\frac{\Delta \left(R_{n}-G\right)}{\Delta +\gamma }$$

where LE_eq_ is the equilibrium latent flux (W s^−1^). For practical conditions, the LE was calculated as:


$$LE=\Omega LE_{eq}+\left(1-\Omega \right)LE_{im}$$

$$\Omega =\frac{\Delta +\gamma }{\Delta +\gamma (1+g_{a}/g_{s})}$$

The relative change in the LE for a prescribed change in g_s_ is calculated using the following equation:


$$\frac{dLE/LE}{dg_{s}/g_{s}}=1-\Omega$$

According to Priestley and Taylor (1972), the Priestley–Taylor coefficient (α) is the ratio of LE to LE_eq_ as follows:


$$\alpha =\frac{LE}{LE_{eq}}=\frac{LE\left(\Delta +\gamma \right)}{\Delta \left(R_{n}-G\right)}$$

An α > 1 indicates wet surfaces where the water supply is sufficient, whereas α ≤ 1 indicates dry surfaces where the water supply is restricted. To avoid spurious values caused by low solar elevation, we used midday data (10:30–14:30) to calculate *g_a_*, *ET_eq_*, *α*, *Ω*, and *g_s_*.

## 3 Results

### 3.1 Biophysical factors

**Figure 3:**
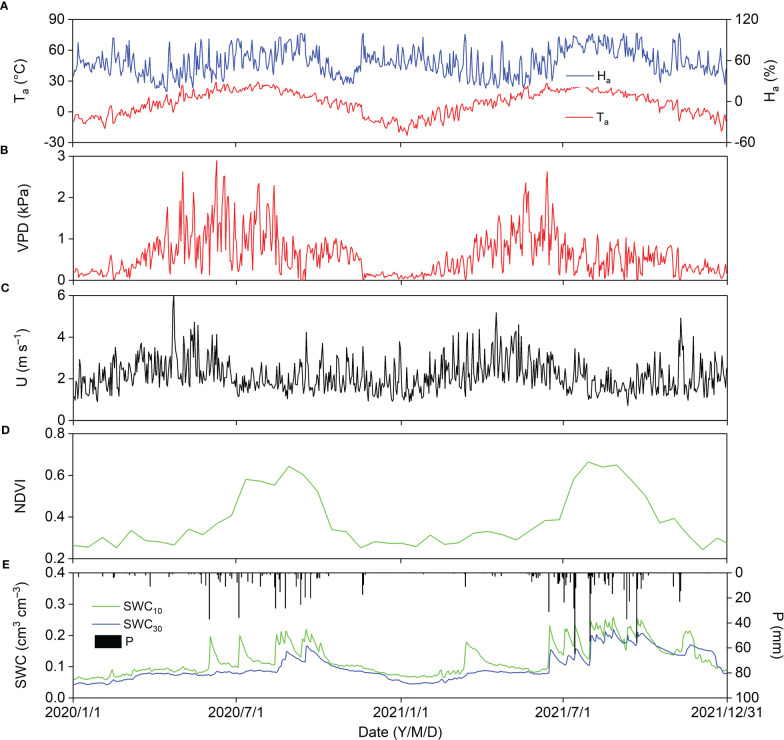
Seasonal variations in biophysical factors in 2020 and 2021; biophysical factors are **(A)** air temperature (T_a_) and relative humidity (H_a_), **(B)** vapor pressure deficit (VPD), **(C)** wind speed (U), **(D)** normalized difference vegetation index (NDVI), and **(E)** precipitation (P), soil water content at 10 cm depth (SWC_10_) and 30 cm depth (SWC_30_).

**Figure 3** shows the seasonal variations in biophysical factors in zthe *Pinus tabuliformis* plantation in 2020 and 2021. Daily T_a_ displayed a parabolic trend, and the lowest and highest T_a_ were −23.08 and 29.05°C, respectively. The daily air relative humidity (H_a_) fluctuated around 50% (**Figure 3A**). The general daily VPD trend decreased notably in 2020 (August 15), two months later in the season than in 2021 (June 15), indicating that the onset of the summer monsoon was earlier in 2020 than in 2021. The average daily VPD was 0.67 and 0.55 in 2020 and 2021, respectively (**Figure 3B**). Daily U usually fluctuated above 1.00 m s^–1^, with a mean value of 2.16 and 2.15 m s^–1^ in 2020 and 2021, respectively (**Figure 3C**). The maximum NDVI, appearing in August, was 0.64 and 0.66 in 2020 and 2021, respectively, with an average of 0.37 in 2020 and 0.39 in 2021, indicating that plant growth was better in 2021 than in 2020 (**Figure 3D**). Annual P in 2020 (435.2 mm) was close to the mean (440 mm), but was much higher in 2021 (632.8 mm), suggesting that 2020 and 2021 were average and wet years, respectively. The seasonal variations in soil water content (SWC) at 10 cm depth (SWC_10_) were strongly dependent on the P pattern, and SWC at 30 cm depth (SWC_30_) increased from August 15 and June 15 in 2020 and 2021, respectively (**Figure 3E**).

### 3.2 Surface energy fluxes

**Figure 4:**
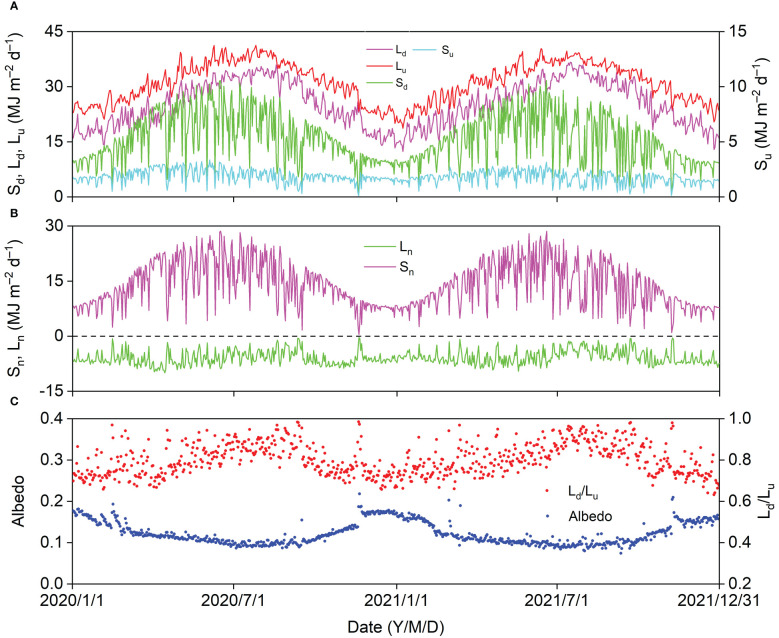
Seasonal variation in **(A)** downward and upward shortwave radiation (S_d_, S_u_), downward and upward longwave radiation (L_d_, L_u_), **(B)** net shortwave and longwave radiation (S_n_, L_n_), and **(C)** albedo (S_u_/S_d_) and L_u_/L_d_ in 2020 and 2021.

Seasonal variations in daily S_d_, S_u_, L_d_, and L_u_ displayed parabolic trends with peak values of 31.17, 3.36, 36.69, and 41.25 MJ d^–1^, respectively (**Figure 4A**). Daily S_d_ showed a similar trend with daily S_n_, and the maximum daily S_n_ was 28.58 MJ d^–1^. Daily L_n_ fluctuated between −0.36 and −10.02 MJ d^–1^ (**Figure 4B**). Daily S_d_, S_u_, L_d_, L_u_, and S_n_ decreased sharply, and daily L_n_ increased sharply on cloudy and rainy days. The daily albedo (S_u_/S_d_) generally decreased from winter to summer with a minimum value of 0.07, which increased sharply with daily S_u_ on some days in winter after a snowfall. The general trend of daily L_d_/L_u_ was contrary to that of albedo, and the day-to-day albedo patterns were relatively more stable (**Figure 4C**).

**Figure 5:**
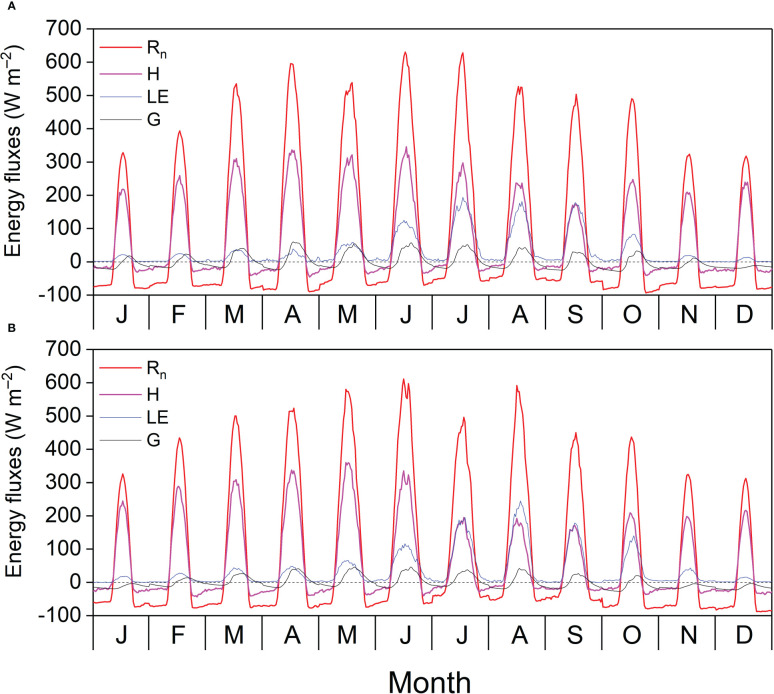
Diurnal cycle of monthly average net radiation (R_n_), sensible heat flux (H), latent heat flux (LE), and soil heat flux (G) in **(A)** 2020 and **(B)** 2021.

Monthly average R_n_ and H exhibited clear diurnal variations and peaked at noon, and the highest diurnal peaks of the monthly average R_n_ (621.07 W s^–1^) and H (360.39 W s^–1^) appeared in June 2020 and May 2021, respectively. The monthly average LE and G displayed clear diurnal variations in the GS and were relatively lower in the DS. The monthly average diurnal courses of G lagged behind those of R_n_, H, and LE and were positive during the day and negative at night because of energy dissipation into and out of the soil, respectively. The most prominent diurnal peaks of the monthly average LE (245.28 W s^–1^) and G (60.06 W s^–1^) appeared in August 2021 and April 2021, respectively (**Figure 5**). **Table A1** shows the characteristics of the energy balance based on the 30-min flux data. The slopes between available energy (R_n_ – G) and turbulent fluxes (LE + H) were 0.71 and 0.73, with the intercepts of 21.70 and 19.01 W m^−2^, and R^2^ values of 0.93 and 0.94 in 2020 and 2021, respectively. The energy balance closure ratios were 0.93 and 0.94 in 2020 and 2021, respectively.

As shown in **Table A2**, albedo in the GS was lower than in the DS, and annual albedo was 0.12 and 0.11 in 2020 and 2021, respectively. Both L_d_/L_u_ and R_n_/(S_d_+L_d_) were higher in the GS than in the DS, with L_d_ offsets of 80% and 82% of L_u_ and R_n_ accounting for 20% and 19% of downward radiation in 2020 and 2021, respectively. In 2020 and 2021, H was slightly greater than R_n_ in the DS, and EF in the GS was 0.34 and 0.38, respectively. In the DS, G was an essential component of the available energy, accounting for –14% and –17% of R_n_ in 2020 and 2021, respectively. The ratio of H to LE was greater than 1 during the different periods in both years, suggesting that H was the major component of R_n_ in this study.

**Figure 6:**
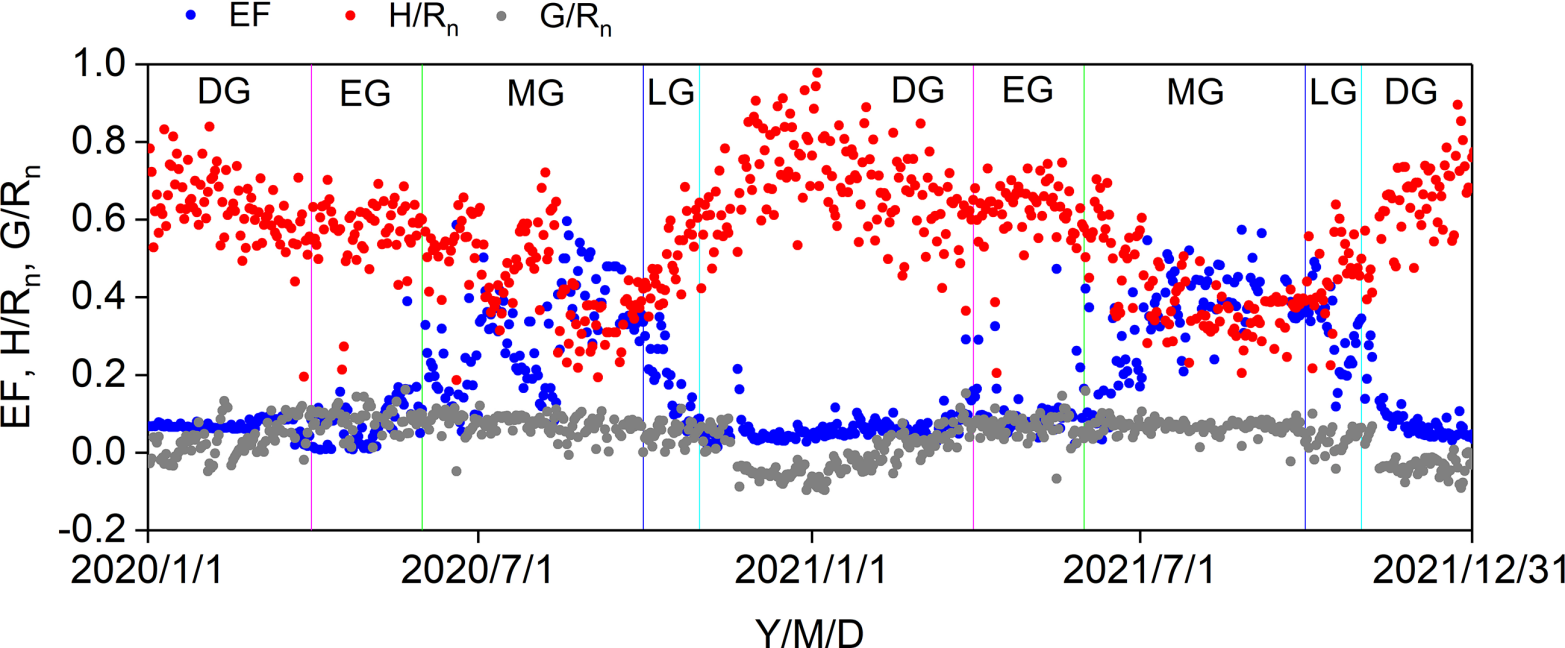
Seasonal variations in midday EF, H/R_n_, and G/R_n_ in 2020 and 2021. DS: dormant season, EG: early growing stage, MG: mid growing stage, LG: later growing stage.

**Figure 7:**
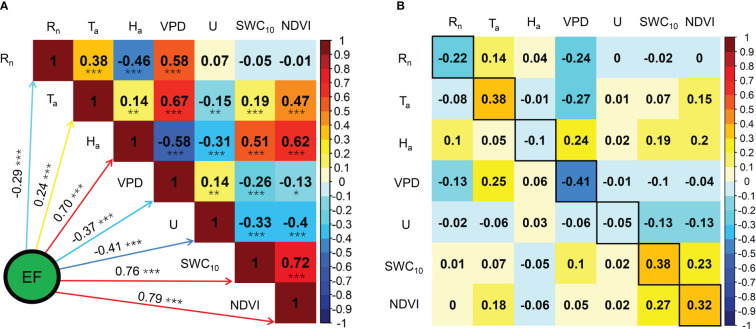
Correlation analysis **(A)** and path analysis **(B)** among midday EF and biophysical factors during the growing seasons in 2020 and 2021. * Significant at p< 0.05; ** significant at p< 0.01; *** significant at p< 0.001.

The seasonal variations in the ratios of midday H, LE, and G to R_n_ are shown in **Figure 6**. Midday G/R_n_ was relatively more stable than midday H/R_n_ and EF and fluctuated between −0.1 and 0.1. Midday H/R_n_ generally decreased until the end of the MG and increased until the end of the year. The minimum midday H/R_n_ was 0.19 in 2020 and 2021. The midday EF generally increased at the beginning of the MG and decreased from the LG, with a maximum midday EF of 0.60. The effects of the biophysical factors on the midday EF are shown in **Figure 7**. All the biophysical factors influenced midday EF at a significance level of p< 0.001, and midday EF was positively correlated with T_a_, H_a_, SWC_10_, and NDVI with correlation coefficient values ranging from 0.24 to 0.79. The remaining factors, namely R_n_, VPD, and U, were negatively correlated with LUE, with correlation coefficient values ranging from −0.29 to −0.41 (**Figure 7A**). A path analysis between the midday EF and biophysical factors showed that the effects of R_n_, T_a_, and VPD on the midday EF were mainly direct. The indirect effects of the remaining factors, namely H_a_, U, SWC_10_, and NDVI, on the midday EF were greater than their direct effects (**Figure 7B**).

### 3.3 Evapotranspiration

**Figure 8:**
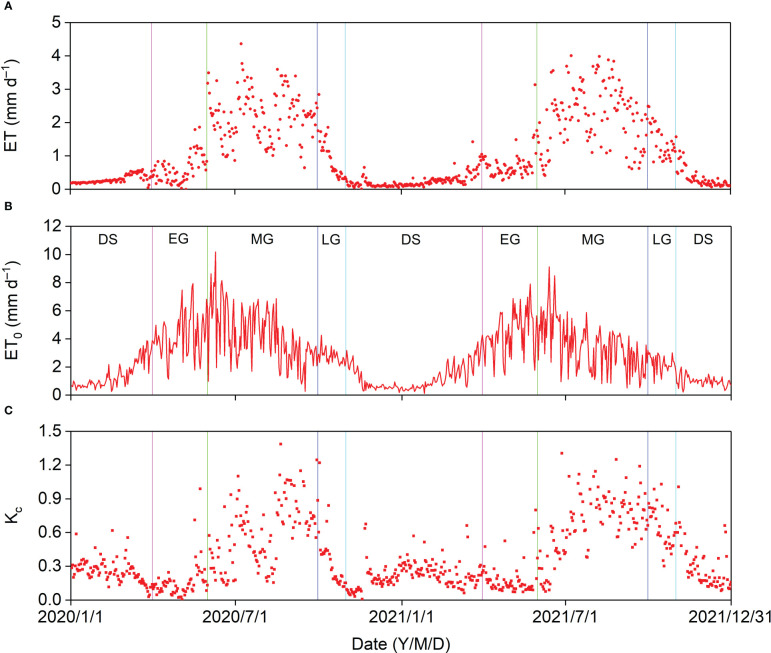
Seasonal variation in **(A)** evapotranspiration (ET), **(B)** reference evapotranspiration (ET_0_), and **(C)** crop coefficient (K_c_) in 2020 and 2021.

The annual ET was 372.37 and 407.66 mm in 2020 and 2021, respectively. The maximum daily ET was 4.36 mm (**Figure 8A**). Daily ET_0_ generally increased before the onset of the MG and decreased after that for the remaining part of the year. However, the daily ET was relatively lower in the DS and EG, increased sharply before the middle of the MG, and decreased after that until the end of the LG (**Figure 8B**). The general daily K_c_ trend was like that of the daily ET. The maximum daily K_c_ was 1.39 and appeared after a P event, and the average daily K_c_ in the MG were 0.60 and 0.68 in 2020 and 2021, respectively (**Figure 8C**).

**Figure 9:**
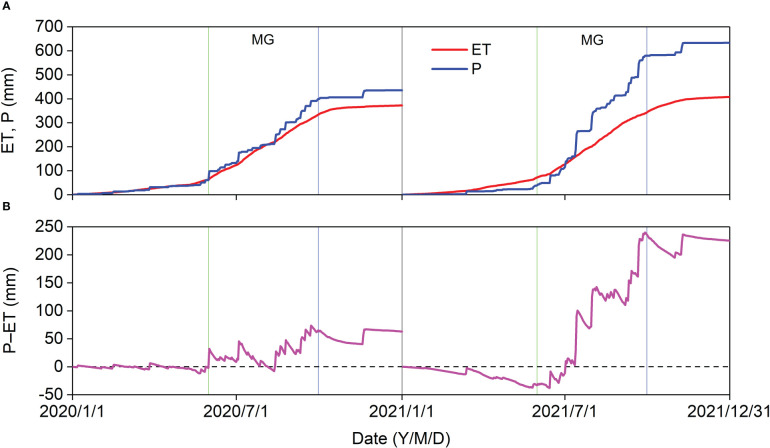
Cumulative **(A)** ET, P, and **(B)** P–ET in 2020 and 2021.

In 2020, the cumulative ET (65.86 mm) was almost equal to the cumulative P (60.07 mm) before the MG, and the cumulative P − ET in the MG (63.24 mm) was almost identical to that in the entire year (62.83 mm) (**Figures 9A, B**). In 2021, the cumulative ET (73.22 mm) was higher than the cumulative P (42.60 mm) before the MG, and the minimum cumulative P − ET (−48.78 mm) almost offsets the cumulative P − ET in 2020. The cumulative P − ET was 239.90 mm in 2021 (**Figures 9A, B**). Thus, 62.83 mm and 239.90 mm of P recharged the soil water and produced surface runoff in 2020 and 2021, respectively. The cumulative ET_0_ (1,044.68 mm in 2020 and 949.32 mm in 2021) was much greater than the cumulative ET and cumulative P, indicating that the *Pinus tabuliformis* plantation was water limited.

**Figure 10:**
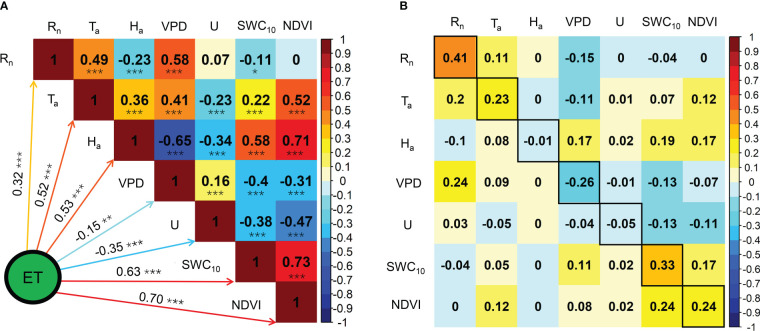
Correlation analysis **(A)** and path analysis **(B)** among daily ET and biophysical factors during the growing seasons in 2020 and 2021. * Significant at p< 0.05; ** significant at p< 0.01; *** significant at p< 0.001.

The effects of biophysical factors on daily ET are displayed in **Figure 10**. The VPD influenced daily ET at a significance level of p< 0.01, and a significance level of p< 0.001 was observed in the remaining biophysical factors. Daily ET was negatively correlated with VPD and U with correlation coefficient values of −0.15 and −0.35, respectively, and R_n_, T_a_, H_a_, SWC_10_, and NDVI were positively correlated with LUE, with correlation coefficient values of 0.32, 0.52, 0.53, 0.63, and 0.70, respectively (**Figure 10A**). Path analysis between daily ET and biophysical factors demonstrated that the effects of R_n_, VPD, and SWC_10_ on daily ET were mainly direct, and the indirect effects of the remaining factors, namely T_a_, H_a_, U, and NDVI, on daily ET were higher than their direct effects (**Figure 10B**).

### 3.4 Surface parameters

**Figure 11:**
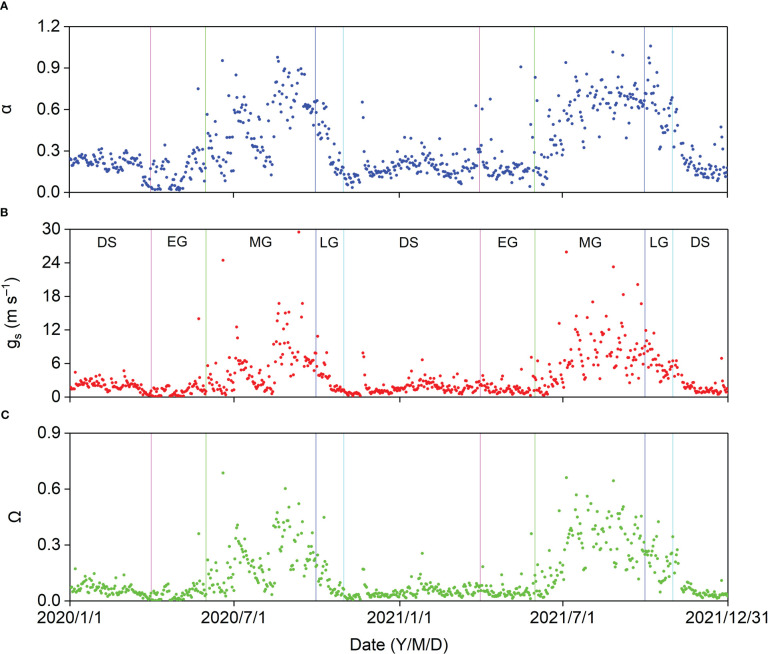
Seasonal variation in midday **(A)** Priestley–Taylor coefficient (α), **(B)** surface conductance (g_s_), and **(C)** decoupling coefficient (Ω) in 2020 and 2021.

The seasonal variations in midday *α*, *g_s_*, and *Ω* are shown in **Figure 11**. These surface parameters increased gradually from the onset of the MG, decreased from the LG, and increased sharply after rainy days. The average midday *α*, *g_s_*, and *Ω* in the different growing stages and DS were higher in 2021 than in 2020 (**Table A3**). The average midday *g_s_* and *Ω* were the highest at 7.20 and 0.29, respectively, in the 2021 MG. The average midday *α* reached the maximum values of 0.51 and 0.63 in the MG of 2020 and in the LG of 2021, respectively, which might be because of the relatively higher ET in the LG of 2021.

**Figure 12:**
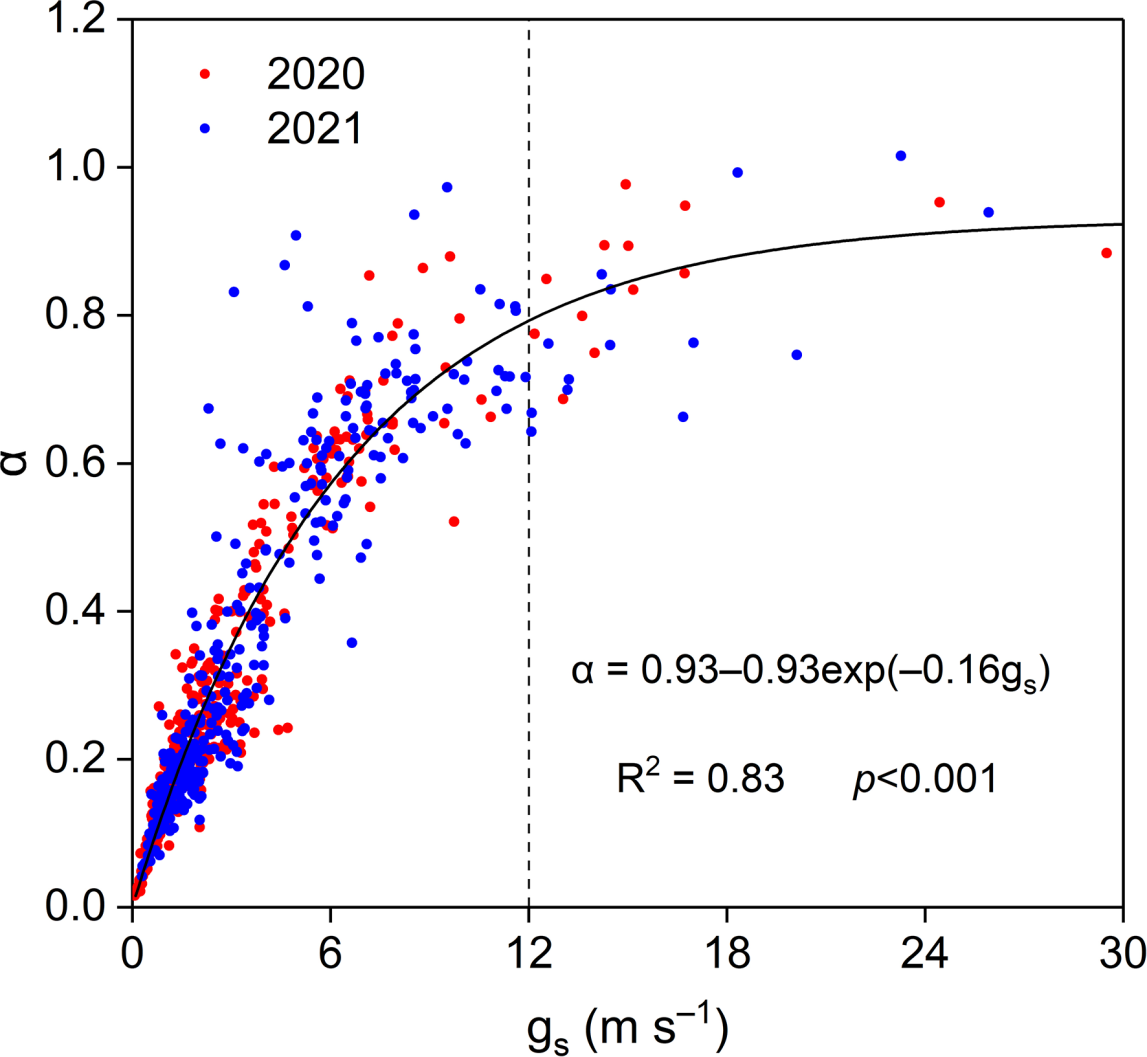
Relationship between midday α and g_s_ in the *Pinus tabuliformis* plantation.

Midday *α* increased exponentially with increasing midday *g_s_*, and midday *α* was insensitive when midday *g_s_* was higher than approximately 12 mm s^−1^, with a midday α asymptotic value of 0.93 (**Figure 12**).

**Figure 13:**
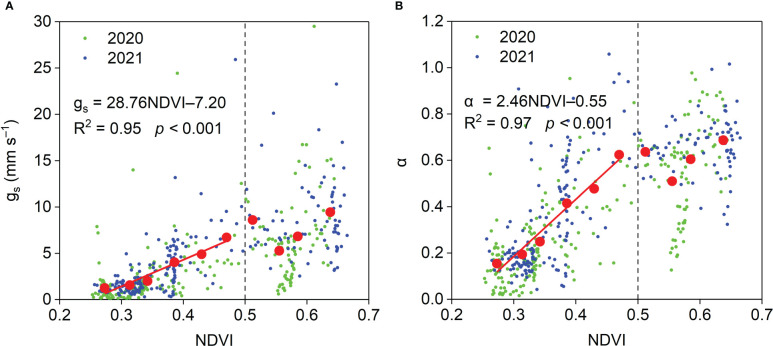
Relationships between midday **(A)** g_s_ and **(B)** α and NDVI and during the growing season in the *Pinus tabuliformis* plantation. Red solid dots: the dependent variables were bin-averaged into 0.04 NDVI increments.

**Figure 14:**
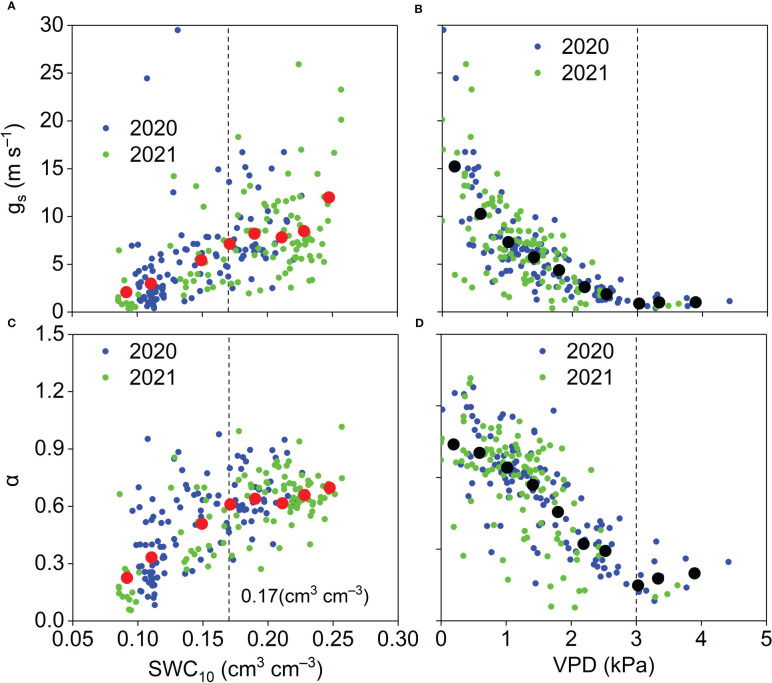
Relationships between midday **(A)** g_s_ and **(B)** α and SWC_10_ and relationships between mid-day **(C)** g_s_ and **(D)** α and VPD and during the growing season in the *Pinus tabuliformis* plantation. Red solid dots: the dependent variables were bin-averaged into 0.02 cm^3^ cm^–3^ SWC_10_ increments **(A, B)**. Black solid dots: the dependent variables were bin-averaged into 0.4 kPa VPD increments **(C, D)**.

The relationships between midday *g_s_* and *α* and NDVI are shown in **Figure 13**. When NDVI was lower than 0.5, midday *g_s_* and *α* per unit NDVI increased by 28.76 mm s^−1^ and 2.46, respectively. When NDVI was higher than 0.5, midday *g_s_* and *α* were insensitive to NDVI. Midday *g_s_* generally increased as SWC_10_ increased (**Figure 14A**). When SWC_10_ was lower than 0.17 cm^3^ cm^−3^, midday *α* generally increased with increasing SWC_10_. When SWC_10_ was higher than 0.17 cm^3^ cm^−3^, midday *α* was insensitive to SWC_10_ (**Figure 14B**). When VPD was lower than 3 kPa, midday *g_s_* and *α* generally decreased with increasing VPD, and when VPD was higher than 3 kPa, midday g_s_ and *α* were insensitive to VPD (**Figures 14C, D**).

## 4 Discussion

### 4.1 Characteristics of surface energy partitioning

In the *Pinus tabuliformis* plantation, the mean annual S_d_, S_u_, L_d_, and L_u_ were 5,754.85, 664.32, 9,219.25, and 11,375.57 MJ, respectively. The S_d_ increased with increasing solar altitude and was dramatically influenced by aerosols and clouds in the atmosphere. Underlying surface characteristics, such as soil moisture, plant canopy, and snow cover, controlled S_u_, which generally depends on S_d_. The annual S_d_ and S_u_ at the study site were comparable to those for a black locust plantation in the Yellow River Delta (Gao et al., 2021). Lower annual S_d_ and higher annual S_u_ were reported in an alpine meadow on the Tibetan Plateau (You et al., 2017), where the latitude is lower, and the air is clearer than at our study site. The annual albedo at our study site was similar to that of a black locust plantation (0.12, Gao et al., 2021) and higher than that in a subalpine spruce forest on the Tibetan Plateau (0.09, Zhu et al., 2013). The annual albedo in an alpine meadow (0.25, You et al., 2017) and farmlands (0.18, Chen et al., 2016; 0.21, Gao et al., 2018) on the Loess Plateau was higher than that for the *Pinus tabuliformis* plantation, which is consistent with the view that forests absorb more solar radiation than other ecosystems. The L_u_ increased with increasing surface temperature, and L_d_ was mainly controlled by the water vapor pressure and T_a_. Annual L_u_ and L_d_ at the study site were higher than those for the Tibetan Plateau (Zhu et al., 2013; You et al., 2017) and comparable to those for the Loess Plateau (Chen et al., 2016; Gao et al., 2018). In addition, the annual L_d_/L_u_ at our site was comparable to that for the two regions (0.76−0.85). Compared with DS, higher L_d_/L_u_ and lower albedo resulted in higher R_n_/(S_d_+L_d_) in GS at our site (**Table A2**), which is consistent with the findings of the previous studies (Zhu et al., 2013; Gao et al., 2018). The annual R_n_/(S_d_+L_d_) at our site was comparable to that of a black locust plantation (0.21, Gao et al., 2021) and higher than that in croplands (0.16, Chen et al., 2016; 0.17, Gao et al., 2018) in northern China.

A comprehensive evaluation of energy balance closure across 22 sites and 50 site years in FLUXNET indicated that the slopes between available energy and turbulent fluxes were between 0.55 and 0.99, the intercepts between −32.9 and 36.9 W m^−2^, the R^2^ values between 0.64 and 0.96, and the energy balance closure ratios between 0.34 and 1.17 (Wilson et al., 2002). Our results are comparable to the FLUXNET results, which indicate that the eddy covariance system provided reliable estimates of turbulent fluxes in the *Pinus tabuliformis* plantation. The energy balance closure ratios were 1.05 and 0.90 in the DS and GS, respectively, indicating that energy balance closure changed with the plant growth at our site. Similar results were observed in an alpine riparian shrubland (Zhang et al., 2014) and three alpine ecosystems of the Qinghai Lake watershed (Zhang et al., 2016) on the Tibetan Plateau.

In the diurnal course of monthly average energy fluxes, LE was close to H in September 2020 and from July to September 2021, and lower than H in the other months (**Figure 5**), which could be because of different quantities of P in the two years (**Figure 3**). In this study, LE was slightly higher than 0 at night, suggesting that the water vapor was lost to the atmosphere, and H was slightly lower than 0 at night, indicating that heat from the atmosphere was used for evaporation and sublimation and offset the heat loss from the plants and soil. A similar phenomenon has been observed in previous studies in various ecosystems (Jia et al., 2016; You et al., 2017; Gao et al., 2018; Gao et al., 2021). The amplitude of diurnal course of monthly average G ranged from 8.45 to 83.97 W m^−2^ in the *Pinus tabuliformis* plantation, which was comparable to a black locust plantation (Gao et al., 2021) and lower than that in rainfed cropland (Gao et al., 2018) and semiarid shrubland in northern China (Jia et al., 2016), which is because G is controlled by R_n_ and plant and litter shade.

The surface energy partitioning in the GS differed from that in the DS in the *Pinus tabuliformis* plantation (**Table A2**), consistent with the results in various ecosystems (Jia et al., 2016; Gao et al., 2018; Gao et al., 2021). In the DS, H/R_n_ was slightly higher than 1, which might be because the measured energy fluxes came from different areas (Wilson et al., 2002), indicating that H was the primary consumer of R_n_ in this study, as observed in various other ecosystems (Kang et al., 2015; Jia et al., 2016; You et al., 2017; Gao et al., 2018). The EF is regarded as an indicator of plant moisture conditions, with an average EF of 0.36 in the GS at our study site (**Table A2**), which is comparable to those of a maritime pine forest (Jarosz et al., 2008) and a black locust plantation (Gao et al., 2021) and lower than those of well-watered forests (Kang et al., 2015; Meijide et al., 2017; Yan et al., 2017; Geng et al., 2020). This indicates that the *Pinus tabuliformis* plantation is a water-limited ecosystem through 2021 was a wet year. The H/LE (i.e., Bowen ratio) is an essential parameter of surface energy fluxes and plays a crucial role in estimating turbulent fluxes using the Bowen ratio-energy balance method. The H/LE was higher than 1 in the GS in this study (**Table A2**), which is consistent with many results reported in arid and semiarid regions (Jia et al., 2016; Yue et al., 2019). Although G is an essential component of the surface energy balance in the DS, G/R_n_ was close to 0 for the entire year in this study (**Table A2**), indicating that the local climate and hydrology were mainly impacted by the partitioning of R_n_ between LE and H over extended timescales. Compared with 2020, EF and H/LE were higher and lower, respectively, in 2021 (**Table A2**). We believe that this phenomenon is mainly because of the much higher P in 2021, which provided more water for the *Pinus tabuliformis* plantation. Previous studies in arid and semiarid regions have also reported different annual P resulting in different annual surface energy partitions (Gao et al., 2018; Yue et al., 2019).

Similar to our results (**Figure 7A**), previous studies reported that midday EF was negatively correlated with VPD and solar radiation and positively correlated with T_a_, SWC_10_, and NDVI in semiarid ecosystems (Aires et al., 2008; Jia et al., 2016). In our study, the effects of H_a_ and U on midday EF were mainly indirect *via* other biophysical factors, particularly VPD, SWC_10_, and NDVI (**Figure 7B**), indicating that the effects of H_a_ and U on midday EF depended on other biophysical factors. The correlation coefficients of SWC_10_ and NDVI on midday EF were the highest among the biophysical factors, indicating that soil moisture and plant growth played crucial roles in the *Pinus tabuliformis* plantation energy partitioning.

### 4.2 Characteristics of ET

Annual ET (372.37 mm in 2020 and 407.66 mm in 2021) at our study site was comparable to that of a typical temperate steppe (Li et al., 2016) and a young plantation (Ma et al., 2018), lower than that of a cork oak plantation (Tong et al., 2014) and a poplar plantation (Kang et al., 2015), and higher than that of semiarid shrubland (Jia et al., 2016) and a temperate desert steppe (Li et al., 2016) in northern China. Annual ET variations at different study sites should be related to the local climate, plant characteristics, and management practices. Annual ET/P is a valuable metric for quantifying the effects of land use and climate change on regional hydrology. Annual ET/P at our site was 0.86 and 0.64 in 2020 and 2021, respectively, comparable to the results in many temperate forests (Li et al., 2016; Ma et al., 2018; Gao et al., 2021). Compared with local natural ecosystems (1.00 for temperate typical steppe and 1.91 for temperate meadow steppe) close to our site (Li et al., 2016), the annual ET/P was lower in our study, suggesting that the *Pinus tabuliformis* plantation did not cause deep soil desiccation and had a positive effect on the hydrological cycle in our study area. However, drier and warmer climates (**Figure 2**) may increase the water requirement of the *Pinus tabuliformis* plantation, and maintaining the hydrological benefit of the plantation in the future will be challenging. As P was relatively low before July 2021, the minimum cumulative P − ET was −48.78 mm in the same period (**Figure 9**), indicating that the plantation consumed the water accumulated in 2020 before the rainy season in 2021. Soil moisture carry-over from previous years is vital for allowing plantations to withstand the dry period before the rainy season in semiarid regions (Ma et al., 2018).

Similar to the findings of previous studies in semiarid ecosystems (Jia et al., 2016; Gao et al., 2018; Yue et al., 2019), plant growth, soil moisture, solar radiation, and temperature had positive effects on daily ET in the *Pinus tabuliformis* plantation (**Figure 10A**). The effects of H_a_ and U on daily ET were mainly indirect *via* other biophysical factors, particularly VPD, SWC_10_, and NDVI (**Figure 10B**), indicating that the effects of H_a_ and U on daily ET also depended on other biophysical factors at our site. In this study, the effect of VPD on daily ET was negative (**Figure 10A**), mainly because the positive effect of VPD on ET could not offset its negative effect on *g_s_*. Similar results were observed in a Mongolian steppe (Li et al., 2007).

The general daily ET_0_ trend was not consistent with daily ET (**Figure 8**), indicating that the seasonal ET pattern in the *Pinus tabuliformis* plantation was controlled by plant phenology and was therefore highly related to plant growth. Similar results were observed in a black locust plantation (Gao et al., 2021) and a riparian *Tamarix* spp. stand in northwest China (Yuan et al., 2014). The K_c_ can be used for the applications related to irrigation planning, and is a crucial parameter for estimating ET in croplands using the single-crop coefficient approach. This parameter has attracted considerable attention in natural ecosystems in recent years because it is an indicator of the plant water status (Yuan et al., 2014; Gao et al., 2018; Gao et al., 2021). The average daily K_c_ in the MG was higher than 0.9 in irrigated croplands in northwest China (Ding et al., 2013; Zhang et al., 2016), which was higher than the results in our study, indicating that the water consumption intensity was relatively low in the *Pinus tabuliformis* plantation.

Many studies have demonstrated that annual ET increases with increasing annual P in arid and semiarid regions (Gao et al., 2018; Yue et al., 2019), and similar results were observed in this study (**Figure 9**). Annual P in 2021 was 45% more than that in 2020, and SWC_30_ in 2021 increased two months earlier than in 2020 (**Figure 3**); however, the annual ET in 2021 was only 9% more than that in 2020. We suggest three reasons for this phenomenon: (1) *Pinus tabuliformis* is a native evergreen coniferous species in the semiarid regions of North China and has a relatively low transpiration rate owing to its resistance to drought; (2) high P resulted in lower solar radiation and VPD, and a lower ET_0_ in 2021; and (3) higher P might produce surface runoff, and annual available water should be lower than annual P in the *Pinus tabuliformis* plantation in 2021.

### 4.3 Characteristics of surface parameters

The seasonal patterns of midday *α*, *g_s_*, and *Ω* in our study were similar to those observed in other ecosystems (Zhu et al., 2013; Gao et al., 2018; Yue et al., 2019). The average midday *α*, *g_s_*, and *Ω* in the MG in this study were higher than those for a semiarid shrubland (Jia et al., 2016) and three Inner Mongolian steppe ecosystems (Chen et al., 2009) with lower ET and lower than those for a hilly tea plantation (Geng et al., 2020) and a conifer and broadleaf mixed forest (Yan et al., 2017) with higher ET. The midday *α* was generally lower than 1 (**Figure 11**), suggesting that the water supply was restricted in the *Pinus tabuliformis* plantation. When Ω is close to 1, R_n_ contributes more to ET, and when Ω is close to 0, g_s_ and VPD contribute more to ET (Monteith and Unsworth, 1990). Midday Ω was generally lower than 0.5 (**Figure 11**), indicating that the underlying surface was well coupled with environmental conditions. The seasonal ET pattern in our study plantation was mainly controlled by g_s_ and VPD.

Midday *α* increased exponentially with midday g_s_ at our study site, indicating strong physiological and phenological regulation of energy partitioning and ET. Similar results have been reported in previous studies (Chen et al., 2009; Jia et al., 2016; Yan et al., 2017; Gao et al., 2018). Midday *α* increased with increasing midday g_s_ until the threshold (ca. 12 mm s^−1^; **Figure 12**), indicating that g_s_ strongly controlled ET when g_s_< 12 mm s^−1^. A theoretical study pointed out that *α* is insensitive to g_s_ when g_s_ > 16 mm s^−1^ in a fully developed canopy (McNaughton and Spriggs, 1986). The asymptotic value of midday α in our study was lower than the reference Priestley–Taylor coefficient of 1.26 (Priestley and Taylor, 1972) and that for well-watered ecosystems (Yan et al., 2017; Geng et al., 2020). The lower threshold value of g_s_ and asymptotic value of α in this study indicated poor soil moisture and an open canopy in the *Pinus tabuliformis* plantation.

Our results, demonstrating that NDVI affected the seasonal variations in midday g_s_ and *α* (**Figure 13**), were consistent with the results in various other ecosystems (Chen et al., 2009; Jia et al., 2016; Gao et al., 2018; Gao et al., 2021). These results indicate that plant growth plays an essential role in surface development at our study site. The midday g_s_ was strongly influenced by SWC_10_ in this study (**Figure 14A**), and similar results were observed in semiarid ecosystems (Aires et al., 2008; Jia et al., 2016). These results indicate that soil moisture has an important effect on stomatal behavior. Like our results (**Figure 14B**), Aires et al. (2008) reported that when surface SWC < 0.15 cm^3^ cm^−3^, midday *α* increased linearly with surface SWC, and when top SWC ≥ 0.15 cm^3^ cm^−3^, midday *α* was insensitive to surface SWC in a Mediterranean grassland in southern Portugal. These results suggest that ET is insensitive to soil moisture when the soil moisture is higher than a specific amount in different ecosystems. A negative effect of VPD on midday g_s_ was observed in this study (**Figure 14C**), which is consistent with the results in water-limited ecosystems (Aires et al., 2008; Zhu et al., 2013; Jia et al., 2016), indicating the importance of biotic regulation on ET. Midday *α* generally decreased linearly with VPD owing to the relationship between midday *α* and g_s_ in this study. When VPD ≥ 3 kPa, midday *α* was insensitive to VPD, perhaps because higher VPD resulted in stomatal closure (**Figure 14D**).

## 5 Conclusion

This 2-year eddy covariance measurements were used to investigate energy and water vapor fluxes over a *Pinus tabuliformis* plantation in Northeast China. The H was the dominant component of R_n_ at all growth stages, and canopy growth and soil water status dominated surface energy partitioning in this plantation. Daily ET was mainly controlled by vegetation development and water availability in our study area, and this plantation had a positive water balance in 2020 (62.83 mm) and 2021 (239.90 mm). Midday α and g_s_ were significantly influenced by NDVI, SWC_10_, and VPD until the NDVI (0.5), SWC_10_ (0.17 mm^3^ mm^−3^), and VPD (3 kPa) thresholds were reached, respectively. This study could enhance our understanding of surface energy partitioning and ET in the vegetation rehabilitation areas of Northeast China.

## Data availability statement

The raw data supporting the conclusions of this article will be made available by the authors, without undue reservation.

## Author contributions

Conceptualization, JZ and XG; methodology, JC and SP; software, LL; validation, HH; writing—original draft preparation, XG; writing—review and editing JZ and XG; visualization, PM; supervision, JZ and XG. All authors contributed to the article and approved the submitted version.

## Appendix

**Table A1:** Characteristics of energy balance and annual ratio of Σ(LE+H)/Σ(R_n_−G) in 2020 and 2021. R_n_: net radiation.

Year	Slope	Intercept (W m^–2^)	R^2^	Annual ratio
2020	0.71	21.70	0.93	0.93
2021	0.73	19.01	0.94	0.94

**Table A2:** Energy partitioning during the growing season, the dormant season, and the entire year in 2020 and 2021.

Year	Period	S_u_/S_d_	L_d_/L_u_	R_n_/(S_d_+L_d_)	H/R_n_	EF	G/R_n_	H/LE
2020	Growing Season	0.11	0.83	0.23	0.53	0.34	0.03	1.56
	Dormant Season	0.15	0.75	0.12	1.02	0.17	-0.14	6.16
	Entire year	0.12	0.80	0.20	0.62	0.31	0.00	2.03
2021	Growing Season	0.10	0.85	0.23	0.50	0.38	0.03	1.32
	Dormant Season	0.14	0.76	0.12	1.01	0.23	-0.17	4.46
	Entire year	0.11	0.82	0.19	0.60	0.35	-0.01	1.70

**Table A3:** Average midday Priestley–Taylor coefficient (α), surface conductance (g_s_), and decoupling coefficient (Ω) during the early growing stage (EG), the mid growing stage (MG), the later growing stage (LG), and the dormant season (DS) in 2020 and 2021.

Year	Period	α	g_s_ (mm s^–1^)	Ω
2020	EG	0.16	1.33	0.04
	MG	0.51	5.73	0.22
	LG	0.33	2.97	0.10
	DS	0.19	1.72	0.05
2021	EG	0.22	1.66	0.06
	MG	0.57	7.20	0.29
	LG	0.63	5.98	0.21
	DS	0.21	1.94	0.06
